# Supplementary material for: Saturation of the compression of two interacting magnetized plasma toroids evidenced in the laboratory
Source: Nat Commun. 2024 Nov 20;15:10065. doi: 10.1038/s41467-024-53938-3 (PMC11579004; doi:10.1038/s41467-024-53938-3)
Supplement: Supplementary file 1 — Supplementary Information [file 41467_2024_53938_MOESM1_ESM.pdf]

Supplemental Material: Saturation of the compression of two interacting magnetized plasma toroids evidenced in the laboratory

A. Sladkov,[1,*] C. Fegan,[2,*] W. Yao,[3,4] A.F.A. Bott,[5] S. N. Chen,[6] H. Ahmed,[7] E.D. Filippov,[8] R. Lelièvre,[3,9] P. Martin,[2] A. McIlvenny,[2,10] T. Waltenspiel,[3,11,12] P. Antici,[12] M. Borghesi,[2] S. Pikuz,[13] A. Ciardi,[4] E. d'Humières,[11] A. Soloviev,[14] M. Starodubtsev,[14] and J. Fuchs[3,†]

[1] *Light Stream Labs LLC, USA, Palo Alto, CA 94306*

[2] *Centre for Light-Matter Interactions, School of Mathematics and Physics, Queen's University Belfast, Belfast BT7 1NN, United Kingdom*

[3] *LULI - CNRS, CEA, UPMC Univ Paris 06 : Sorbonne Université, Ecole Polytechnique, Institut Polytechnique de Paris - F-91128 Palaiseau cedex, France*

[4] *Sorbonne Université, Observatoire de Paris, Université PSL, CNRS, LERMA, F-75005, Paris, France*

[5] *Department of Physics, University of Oxford, Parks Road, Oxford OX1 3PU, United Kingdom*

[6] *ELI-NP, "Horia Hulubei" National Institute of Physics and Nuclear Engineering, Bucharest - Magurele, Romania*

[7] *STFC, U.K.*

[8] *CLPU, 37185 Villamayor, Spain*

[9] *Laboratoire de micro-irradiation, de métrologie et de dosimétrie des neutrons, PSE-Santé/SDOS, IRSN, 13115 Saint-Paul-Lez-Durance, France*

[10] *Lawrence Berkeley National Laboratory, 1 Cyclotron Road, Berkeley, CA, USA*

[11] *University of Bordeaux, Centre Lasers Intenses et Applications, CNRS, CEA, UMR 5107, F-33405 Talence, France*

[12] *INRS-EMT, 1650 boul, Lionel-Boulet, Varennes, QC, J3X 1S2, Canada*

[13] *HB11 Energy Holdings, Freshwater, NSW 2096, Australia*

[14] *Independent Researcher*

(Dated: October 1, 2024)

Figure S1 shows similar results as in Fig.2 of the main paper, but for another separation between the targets, being here $\alpha = 400$ μm. It confirms the robustness of the observed magnetic field compression.

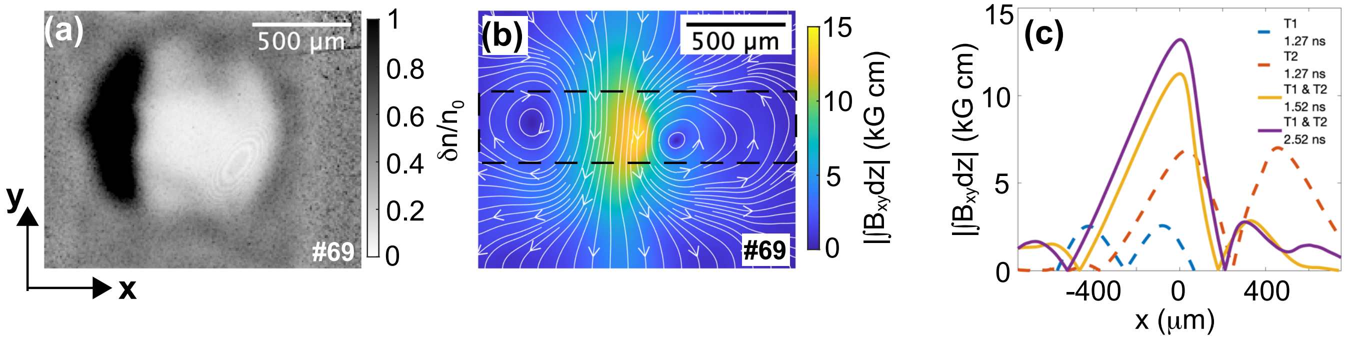

FIG. S1. (a) Experimental proton-radiography image (see Methods) probing the magnetic fields in the plasma plumes produced when targets T1 and T2 are both in place. The image is a snapshot taken at at 2.52 ns, with $\alpha = 400$ μm). (b) Resulting path-integrated magnetic field strength analyzed via the code PROBLEM [1]. The white-arrow streamlines represent the in-plane magnetic field lines (B_x and B_y), and the colormap shows the path-integrated (along the z-axis) strength of the xy-plane magnetic field. (c) Lineout, along the z-axis, of the path-integrated magnetic field strength (measured in the black dashed box shown in panel (b) and averaged along the y-axis). Also shown are the same lineouts obtained also for $\alpha = 400$ μm but for a shot taken at an earlier time (shot 68 at 1.52 ns), as well as the single target shots already shown in the main paper (in panels (a1-a2) of Fig.2, i.e. shots 14 and 15).

TABLE S1. The table not only details the parameters of the magnetic compressed sheet of the laboratory experiment (present case), but it also details those retrieved from the satellite observations of an interaction between coronal mass ejecta [2] and of colliding supernova remnants [3]. We can see that in all cases, the Reynolds number (the ratio of the convection over ohmic dissipation) as well as the magnetic Reynolds number (the ratio of the convection over magnetic diffusion) and the Peclet number (the ratio of magnetic convection over magnetic diffusion) are larger than one.

	Experiment	Scolini, et al., (2020)	Williams, et al., (1997)
Inflow velocity V (km/s)	50	250	25
Temperatures T (eV)	17	100	800
Mass number A	64	1	1
Charge number Z	6	1	1
Sound velocity C_s (km/s)	17	180	510
Alfven velocity V_A (km/s)	5.6	150	11
Local magnetic field B_0 (T)	450	3e-8	2.5e-9
Upstream magnetic field B_{up} (T)	60	1e-8	1.8e-9
Electron density (cm^{-3})	1.3e21	2	7
Sound Mach number (M_s)	3	1.4	0.05
Alfven Mach number (M_A)	10	1.6	2.3
Characteristic spatial scale L (cm)	3e-2	1.5e12	4.6e19
Reynolds number (R_e)	1.2e3	5.6e5	1.4e10
Magnetic Reynolds number (R_m)	63	1.5e17	1e25
Peclet number (P_e)	3.5	1.3e4	3.3e8
Measured magnetic compression ratio	8	2.8	1.4
Expected magnetic compression ratio	7	3.2	2.3
Thermal β	0.1	0.18	1e3
Dynamic β	0.7	0.58	5

REFERENCE

* These two authors contributed equally
† julien.fuchs@polytechnique.edu

[1] A. Bott, C. Graziani, P. Tzeferacos, T. White, D. Lamb, G. Gregori, and A. Schekochihin, Proton imaging of stochastic magnetic fields, Journal of Plasma Physics **83**, 905830614 (2017).

[2] C. Scolini, E. Chané, M. Temmer, E. K. J. Kilpua, K. Dissauer, A. M. Veronig, E. Palmerio, J. Pomoell, M. Dumbović, J. Guo, L. Rodriguez, and S. Poedts, Cme–cme interactions as sources of cme geoeffectiveness: The formation of the complex ejecta and intense geomagnetic storm in 2017 early september, The Astrophysical Journal Supplement Series **247**, 21 (2020).

[3] R. M. Williams, Y. Chu, J. R. Dickel, R. Beyer, R. Petre, R. C. Smith, and D. K. Milne, Supernova remnants in the magellanic clouds. i. the colliding remnants dem l316, The Astrophysical Journal **480**, 618–632 (1997).
